# Retraction: Purification, characterization, and hydrolysate analysis of dextranase from *Arthrobacter oxydans* G6-4B

**DOI:** 10.3389/fbioe.2025.1702209

**Published:** 2025-09-19

**Authors:** Frontiers Editorial Office

The Journal retracts the 10 February 2022 article cited above.

Following publication, the publisher found evidence of an undisclosed conflict of interest that undermined the integrity of the peer review process, and impacted peer reviewer recommendations. Post-publication assessment of the article confirmed that the article does not meet the standards of the journal. As the scientific integrity of the article cannot be guaranteed, and in adherence to the recommendations of the Committee on Publication Ethics (COPE), the article is retracted.

This retraction was approved by the Chief Executive Editor of Frontiers. The authors received a communication regarding the retraction and had a chance to respond. This communication has been recorded by the publisher.

